# Two new species of *Exidia sensu lato* (Auriculariales, Basidiomycota) based on morphology and DNA sequences

**DOI:** 10.3389/fmicb.2022.1080290

**Published:** 2023-02-14

**Authors:** Ablat Tohtirjap, Shi-Xing Hou, Bernard Rivoire, Genevieve Gates, Fang Wu, Yu-Cheng Dai

**Affiliations:** ^1^Institute of Microbiology, School of Ecology and Nature Conservation, Beijing Forestry University, Beijing, China; ^2^Administration of Yuyuantan Park, Beijing, China; ^3^Retired, Orliénas, France; ^4^Tasmanian Institute of Agriculture, Hobart, TAS, Australia

**Keywords:** Auriculariaceae, phylogenetic analysis, taxonomy, wood-rotting fungi, diversity

## Abstract

In the present study, fourteen *Exidia*-like specimens were collected from China, France, and Australia. Based on morphological characteristics and phylogenetic analyses using the internal transcribed spacer regions (ITS) and the large subunit of nuclear ribosomal RNA gene (nLSU), four species in *Exidia sensu lato*, including *Exidia saccharina* and *Tremellochaete atlantica*, and two new species, *Exidia subsaccharina* and *Tremellochaete australiensis*, were identified. The four species are described and illustrated in detail. *E. saccharina* and *T. atlantica*, two known species from China are reported for the first time. *E. subsaccharina* and *T. australiensis*, two new species from France and Australia, respectively are also described. *E. subsaccharina* is characterized by its reddish brown to vinaceous brown basidiomata, slightly papillate hymenial surface, and narrowly allantoid basidiospores without oil drop measuring 12.5–17.5 × 4.2–5.5 μm. It differs from the similar species, *E. saccharina*, by distinctly larger basidiospores (12.5–17.5 × 4.2–5.5 vs. 10–14.2 × 3.2–4.5 μm). *Tremellochaete australiensis* is characterized by its white to grayish blue basidiomata, obviously and densely papillate hymenial surface, and allantoid basidiospores with oil drop measuring 13.8–16.2 × 4.8–6.5 μm. It also can be distinguished from the similar species, *T. atlantica* and *T. japonica*, by its distinctly larger basidiospores (13.5–17.8 × 4–5.2 vs. 10–11.8 × 4–4.8 μm in *T. atlantica*; 9.4–11.8 × 3.5–4.2 μm in *T. japonica*).

## Introduction

*Exidia* Fr. was proposed by Fries and typified by *Exidia glandulosa* (Bull.) Fr. The genus is characterized by gelatinous bassidiomata, ellipsoid to subglobose, longitudinally cruciate septate, 4-celled basidia, cylindrical to allantoid basidiospores, and the ability to cause white rot in woody plants (Lowy, 1971; Liu, 1992; Roberts, 2001; Spirin et al., 2018; Ye et al., 2020; Wu et al., 2022a). Because of their morphological similarities, *Exidia sensu lato* traditionally includes three genera *Exidia, Myxarium* Wallr., and *Tremellochaete* Raitv (Roberts, 1998; Weiß and Oberwinkler, 2001; Malysheva, 2012), as confirmed by phylogenetic analyses in recent studies (Malysheva and Spirin, 2017; Spirin et al., 2018, 2019a; Wu et al., 2020a). Some species in *Hyaloria* Möller, *Stypella* Möller, and Sebacina C. Tul and C. Tul. et al., have waxy, very small, and effused basidiomata different from the usually gelatinous, thick, orbicular basidiomata of *Exidia*, were recently transferred into *Myxarium* based on morphological and phylogenetic analyses (Spirin et al., 2018). *Myxarium* phylogenetically forms a monophyletic clade distantly related to *Exidia* and *Tremellochaete* (Spirin et al., 2019a; Stalpers et al., 2021). In addition, *Myxarium* belongs to Hyaloriaceae, whereas *Exidia* and *Tremellochaete* belong to Auriculariaceae, and *Myxarium* can be distinguished from the latter two genera by its distinctly stalked basidia (Spirin et al., 2018, 2019a). Therefore, *Exidia sensu lato* is defined here as a group of fungi that includes *Exidia* and *Tremellochaete*.

*Exidia* was less studied in the latter part of the 20th century but has received attention more recently (Weiß and Oberwinkler, 2001; Wells et al., 2004; Roberts, 2009) due to its edible species and medicinal values (Łopusiewicz, 2018; Wu et al., 2020a). One edible species from China, *Exidia yadongensis* described by F. Wu et al., contains rich amino acids and plays a key role in the balance of physiological functions (Chen et al., 2019; Wu et al., 2020a). Recently, *E. reflexa* F. Wu et al., *E. subglandulosa* F. Wu et al., and *E. qinghaiensi* S.R. Wang and Thorn, were described based on multigene phylogenies (Ye et al., 2020; Wang and Thorn, 2021). However, the genus is still polyphyletic in the phylogeny, and species of the genus are scattered in several genera of Auriculariaceae (Yuan et al., 2018; Spirin et al., 2019a,b; Ye et al., 2020). *Tremellochaete* was reinstated to accommodate *T. japonica* (Yasuda), Raitv. and *T. nigerrima* (Viégas), Spirin and Malysheva (Malysheva and Spirin, 2017), and *T. atlantica* Alvarenga and *T. cerradensis* Alvarenga, and one new combination species, *T. ciliata* (Möller) Spirin and Alvarenga, were described and proposed in this genus (Alvarenga et al., 2019; Phookamsak et al., 2019). In total, there are more than 70 species in *Exidia* and six species in *Tremellochaete* worldwide according to Index Fungorum (http://www.indexfungorum.org) and MycoBank (https://www.mycobank.org), but < 20 species have molecular data (Alvarenga et al., 2019; Wu et al., 2020a; Wang and Thorn, 2021). *Tremellochaete* was reinstated at the genus level, it is not accepted by some researchers (Wang and Thorn, 2021), and the generic demarcation of *Exidia* and *Tremellochaete* is unclear both in morphology and phylogeny (Alvarenga et al., 2019; Ye et al., 2020). Further studies are urgently needed based on more samples and taxa.

In the present study, fourteen *Exidia*-like specimens were collected from China, France, and Australia. After morphological examinations and phylogenetic analyses using the internal transcribed spacer regions (ITS) and the large subunit of the nuclear ribosomal RNA gene (nLSU), four species were identified in *Exidia* and *Tremellochaete*, among which two are new to science, and a detailed description of these species is given in the present study.

## Materials and methods

### Morphology

The studied specimens were deposited at the herbarium of the Institute of Microbiology, Beijing Forestry University (BJFC), with color terms following those outlined by Petersen (1996). Sections mounted in 5% KOH and 2% phloxine B (C_20_H_2_Br_4_C_l4_Na_2_O_5_) were studied at a magnification of up to 1,000 × using a Nikon Eclipse 80i microscope and phase contrast illumination. A Nikon Digital Sight DS-L3 camera was used to photograph microscopic structures. We also used other reagents, including Cotton Blue and Melzer's reagent to observe micromorphology following Wu et al. (2022b). To show the variation in spore sizes, 5% of measurements were excluded from each end of the range and shown in parentheses. At least thirty basidiospores from each specimen were measured. Stalks were excluded from basidia measurements, and the hilar appendage was excluded from basidiospore measurements. The following abbreviations were used: KOH, potassium hydroxide (5%); L, mean length (arithmetic average of all basidiospores length); W, mean width (arithmetic average of all basidiospores width); Q, L/W ratio for each specimen studied; n (a/b), number of basidiospores (a) measured from a given number of specimens (b).

### DNA extraction, PCR reaction, and sequencing

DNA was extracted from dried specimens using a rapid plant genome extraction kit (Aidlab Biotechnologies Co., Ltd., Beijing, China) and modified following Wu et al. (2021). The internal transcribed spacer regions (ITS) and the large subunit of the nuclear ribosomal RNA gene (nLSU) were amplified with primer pairs ITS 4 and ITS 5 (White et al., 1990) and LR0R and LR7 (Vilgalys and Hester, 1990), respectively. The PCR (polymerase chain reaction) procedure for ITS was initial denaturation at 95°C for 3 min, followed by 35 cycles at 94°C for 40 s, 58°C for 45 s, and 72°C for 1 min, and a final extension at 72°C for 10 min. The PCR procedure for nLSU was initial denaturation at 94°C for 1 min, followed by 35 cycles at 94°C for 1 min, 48°C for 1 min, and 72°C for 1.5 min, and a final extension at 72°C for 10 min (Wu et al., 2018). The PCR products were purified and sequenced at the BGI (Beijing Genomics Institute, China), with the same primers that are used in the PCR reactions. The nLSU sequences were obtained by splicing bidirectional sequences because LR0R-LR7 is >1,000 bp.

### Phylogenetic analyses

The new sequences generated in this study and reference sequences retrieved from GenBank (Table 1) were aligned with MAFFT (version 7; Katoh and Standley, 2013) and then manually adjusted in BioEdit and Mesquite version 3.04 software (Hall, 1999; Maddison and Maddison, 2017). A dataset composed of concatenated ITS+nLSU sequences was used in the phylogenetic analyses using the maximum likelihood (ML), maximum parsimony (MP), and Bayesian inference (BI) methods. *Bourdotia galzinii* (Bres.) Trotter was selected as the outgroup in the phylogenetic analyses because the species was closer to species of Auriculariaceae than others but not closely related to species in *Exidia sensu lato* (Spirin et al., 2019a). Except for the outgroup, sequences from the other eleven Auriculariales genera were added to the phylogenetic analyses because *Exidia* was previously shown to be polyphyletic (Yuan et al., 2018; Spirin et al., 2019a).

**Table 1:** Taxa information and GenBank accession numbers used in this study.

**Species**	**Sample**	**GenBank Accession nos**.		**Country**
		**ITS**	**nLSU**	
*Adustochaete nivea*	RLMA 531	MN165954	MN165989	USA
*Adustochaete interrupta*	LR 23435	MK391518	MK391527	Mexico
*Adustochaete rava*	KHL 15526	MK391517	MK391526	Brazil
*Amphistereum leveilleanum*	FP1 06715	KX262119	KX262168	USA
*Amphistereum schrenkii*	HHB 8476	KX262130	KX262178	USA
*Auricularia auricula-judae*	JT 04	KT152099	KT152115	UK
*Auricularia auricula-judae*	Dai 16353	MZ618932	MZ669900	France
*Auricularia cornea*	Dai 13621	MZ618936	MZ669905	China
*Auricularia tibetica*	Dai 13336	MZ618943	MZ669915	China
*Elmerina cladophora*	Otto Miettinen X1902	MG757509	MG757509	Indonesia
*Elmerina efibulata*	Dai 9322	JQ764669	JQ764647	China
*Elmerina sclerodontia*	Otto Miettinen X3269	MG757512	MG757512	Malaysia
*Eichleriella alliciens*	HHB 7194	KX262120	KX262169	USA
*Eichleriella flavida*	LR 49412	KX262137	KX262185	UK
*Eichleriella sicca*	OM 17349	KX262143	KX262191	USA
*Exidia candida*	VS 3921	KY801867	KY801892	Russia
*Exidia candida*	VS 8588	KY801870	KY801895	USA
*Exidia candida*	LE 313211	KY801868	KY801893	Russia
*Exidia candida*	LE 38198	KY801871	KY801896	Russia
*Exidia crenata*	Dai 19464	MT663359	MT664778	Canada
*Exidia crenata*	Wu 26	MT663361	MT664780	Canada
*Exidia glandulosa*	MW 355	AF291273	AF291319	Germany
*Exidia glandulosa*	TUFC 34008	AB871761	AB871742	Japan
*Exidia glandulosa*	Dai 18024	MH213394	MH213426	China
*Exidia glandulosa*	Wu 265	MN850376	MN850356	China
*Exidia pithya*	MW 313	AF291275	AF291321	Germany
*Exidia qinghaiensis*	HMAS 156328	MW353409	MW353409	China
*Exidia qinghaiensis*	HMAS 156376	MW353408	MW353408	China
*Exidia recisa*	MW 315	AF291276	AF291322	Germany
*Exidia recisa*	SL 180317	MT663365	MT664783	Finland
*Exidia reflexa*	Dai 20833	MN850386	MN850362	China
*Exidia reflexa*	Dai 20861	MN850388	MN850364	China
*Exidia reflexa*	Dai 20874	MN850389	MN850365	China
*Exidia repanda*	LY BR 7046	MT663367	MT664784	France
*Exidia saccharina*	Roki 88	AF291277	AF291323	Germany
***Exidia saccharina***	**Dai 15848**	**OP605366**	**OP605350**	**China**
***Exidia saccharina***	**Dai 15890**	**OP605367**	**OP605351**	**China**
***Exidia saccharina***	**Dai 21719**	**OP605368**	**OP605352**	**China**
***Exidia saccharina***	**Dai 21720**	**OP605369**	**OP605353**	**China**
*Exidia subglandulosa*	Wu 270	MN850381	MN850357	China
*Exidia subglandulosa*	Wu 272	MN850383	MN850359	China
*Exidia subglandulosa*	Wu 278	MN850385	MN850361	China
***Exidia subsaccharina***	**Dai 22195**	**OP605370**	**OP605354**	**France**
***Exidia subsaccharina***	**Dai 22187**	**OP605371**	**OP605355**	**France**
*Exidia thuretiana*	Spirin 9999	KY801878	KY801905	Finland
*Exidia thuretiana*	MW 373	AF291278	AF291324	Germany
*Exidia thuretiana*	VS 11185	KY801889	KY801914	Norway
*Exidia truncata*	MW 365	AF291279	AF291325	Germany
*Exidia truncata*	Dai 21231	MT663369	MT664785	Finland
*Exidia uvapassa*	TUFC 34007	AB871863	AB871744	Japan
*Exidia uvapassa*	AFTOL-ID 461	DQ241776	AY645056	Japan
*Exidia yadongensis*	Dai 17209	MT663370	MT664786	China
*Exidia yadongensis*	Dai 17212	MT663373	MT664789	China
*Exidia yadongensis*	Dai 17268	MT663375	MT664791	China
*Exidiopsis calcea*	MW 331	AF291280	AF291326	Germany
*Exidiopsis effusa*	OM 19136	KX262145	KX262193	Finland
*Exidiopsis grisea*	RoKi 162	AF291281	AF291328	Germany
*Grammatus labyrinthinus*	Yuan 1600	KM379139	KM379140	China
*Grammatus semis*	OM10618	KX262146	KX262194	China
*Heteroradulum adnatum*	LR 23453	KX262116	KX262165	Mexico
*Heteroradulum deglubens*	LE 38182	KX262112	KX262162	Sweden
*Heteroradulum deglubens*	Solheim 1864	KX262133	KX262181	Norway
*Heteroradulum kmetii*	VS 6466	KX262104	KX262152	Russia
*Heteroradulum kmetii*	He 4915	MH178262	MH178286	China
*Proterochaete adusta*	VS 9021	MK391520	MK391528	Canada
*Tremellochaete atlantica*	URM 90198	MG594382	MG594384	Brazil
*Tremellochaete atlantica*	URM 90199	MG594381	MG594383	Brazil
***Tremellochaete atlantica***	**Dai 22363**	**OP605374**	**OP605358**	**China**
***Tremellochaete atlantica***	**Dai 22375**	**OP605375**	**OP605359**	**China**
***Tremellochaete atlantica***	**Wu 539**	**OP605373**	**OP605357**	**China**
***Tremellochaete australiensis***	**Dai 18601A**	**OP605376**	**OP605360**	**Australia**
***Tremellochaete australiensis***	**Dai 18664**	**OP605377**	**OP605361**	**Australia**
***Tremellochaete australiensis***	**Dai 18704**	**OP605378**	**OP605362**	**Australia**
***Tremellochaete australiensis***	**Dai 18714**	**OP605379**	**OP605363**	**Australia**
***Tremellochaete australiensis***	**Dai 18758**	**OP605380**	**OP605364**	**Australia**
*Tremellochaete cerradensis*	URM 90200	MK391524	MK391530	Brazil
*Tremellochaete ciliata*	SP 467241	MK391523	MK391529	Brazil
*Tremellochaete japonica*	TAA 42689	AF291274	AF291320	Russia
*Tremellochaete japonica*	Wu 251	MN850378	MN850367	China
*Tremellochaete japonica*	Wu 254	MN850379	MN850368	China
*Bourdotia galzinii* (out group)	Otto MiettinenX3067	MG757511	MG757511	Spain

New sequences are in bold.

Maximum likelihood (ML), Bayesian inference (BI), and maximum parsimony (MP) phylogenetic analyses were performed using RAxML (version 8; Stamatakis, 2014), MrBayes (version 3.2.7a; Ronquist et al., 2012), and PAUP (version 4.0b10; Swofford, 2002), respectively, following the study of Wu et al. (2020b). The optimal substitution models for the combined dataset are determined using the Akaike information criterion (AIC) implemented in MrModeltest 2.3 (Posada and Crandall, 1998; Nylander, 2004) after scoring 24 models of evolution by PAUP (version 4.0b10; Swofford, 2002). The GTR + I + G model was applied in the BI and ML analyses.

Branches that received bootstrap support for maximum likelihood (BS), Bayesian posterior probabilities (BPP), and maximum parsimony (BP) >50% (BS), 0.90 (BPP), and 50% (BP), respectively, are considered to be significantly supported. The phylograms were viewed using FigTree version 1.4.2 (Rambaut, 2012).

## Results

### Phylogenetic analyses

**Figure 1:**
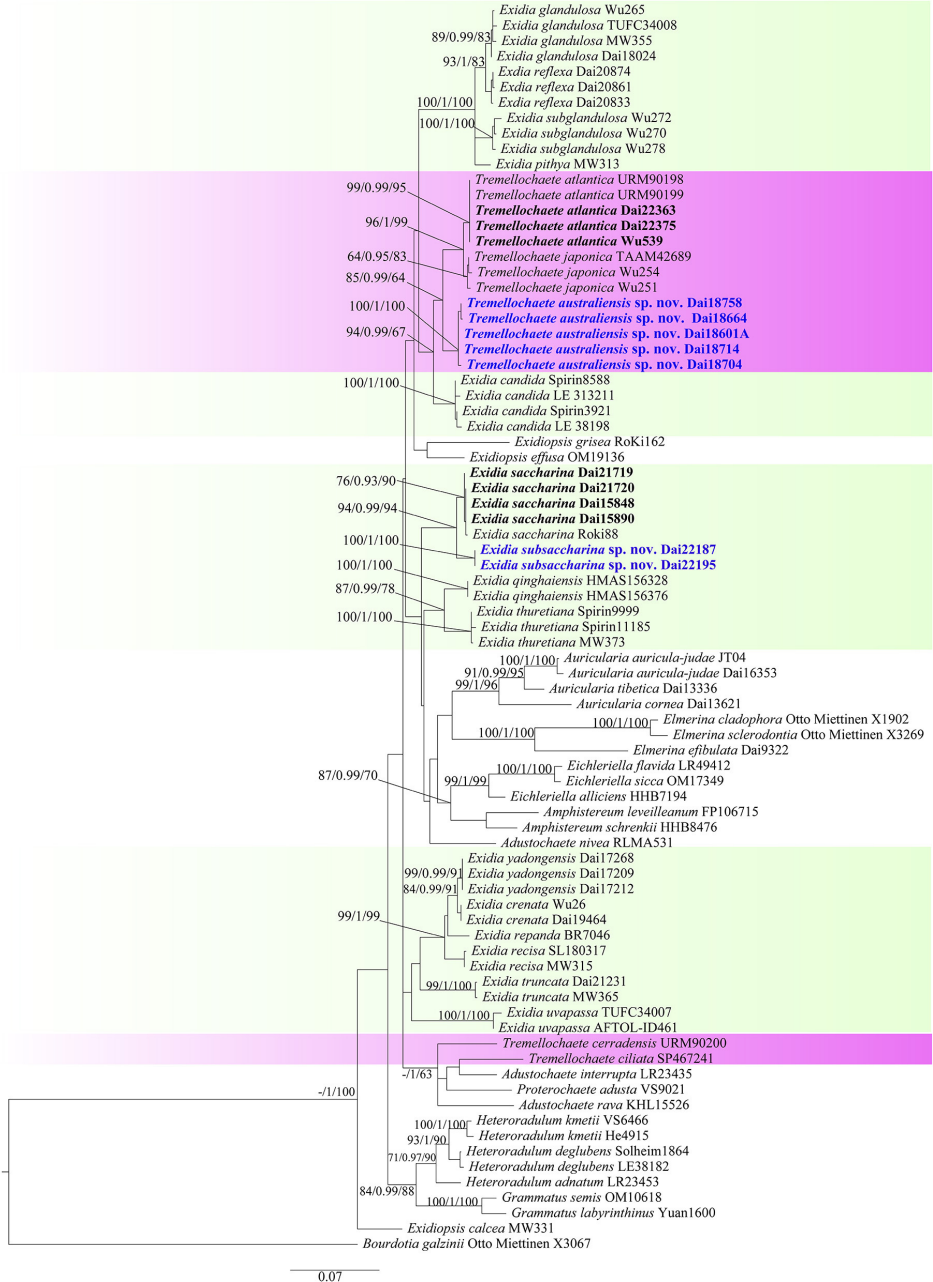
Maximum likelihood tree illustrating the phylogeny of *Exidia sensu lato* based on the combined ITS+nLSU dataset. Branches are labeled with maximum likelihood bootstrap >50%, Bayesian posterior probabilities >0.90, and maximum parsimony bootstrap >50%, respectively. New species are in blue.

The combined ITS+nLSU dataset included 81 fungal specimens representing 44 species in the Auriculariales. The dataset had an aligned length of 1,898 characters, including 1,406 constants, 156 parsimony-uninformative characters, and 336 parsimony-informative characters. MP analysis yielded four equally parsimonious trees (tree length = 1,607, consistency index = 0.432, retention index = 0.725, rescaled consistency index = 0.313, and homoplasy index = 0.568). The average standard deviation of split frequencies in BI analysis was 0.005425. The topology of the ML tree with bootstrap values for BP, BS, and BPP was chosen to represent the phylogenetic relationship of species in the Auriculariales since ML, MP, and BI resulted in similar topologies (Figure 1). The phylogeny demonstrated our fourteen *Exidia*-like specimens were clustered into four different lineages with high support, including two new lineages that represented two new species, *E. subsaccharina* (100% BS, 1.00 BPP, and 100% BP; Figure 1) and *Tremellochaete australiensis* (100% BS, 1.00 BPP, and 100% BP; Figure 1). The four Chinese specimens and one German sample (Roki 88) identified as *E. saccharina* by Weiß and Oberwinkler (2001) were nested in the same lineage with high support in the phylogeny (Figure 1), so these specimens were treated as *E. saccharina*, and this represents the first record of the species in China.

### Taxonomy

***Exidia saccharina*** Fr., Syst. mycol. (Lundae) 2(1): 225 (1822), Figures 2A, 3.

**Figure 2:**
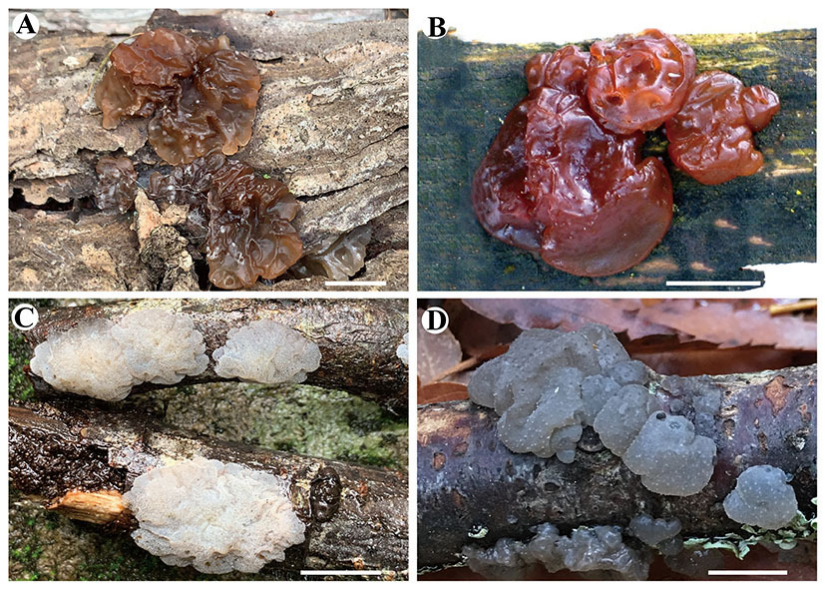
Basidiomata of species in *Exidia* and *Tremellochaete*. **(A)** *Exidia saccharina* (Dai 21719); **(B)** *E. subsaccharina* (Dai 22187 and LY BR 337, holotype); **(C)** *Tremellochaete atlantica* (Dai 22375); **(D)** *T. australiensis* (Dai 18664, holotype). Scale bars: 1 cm.

**Figure 3:**
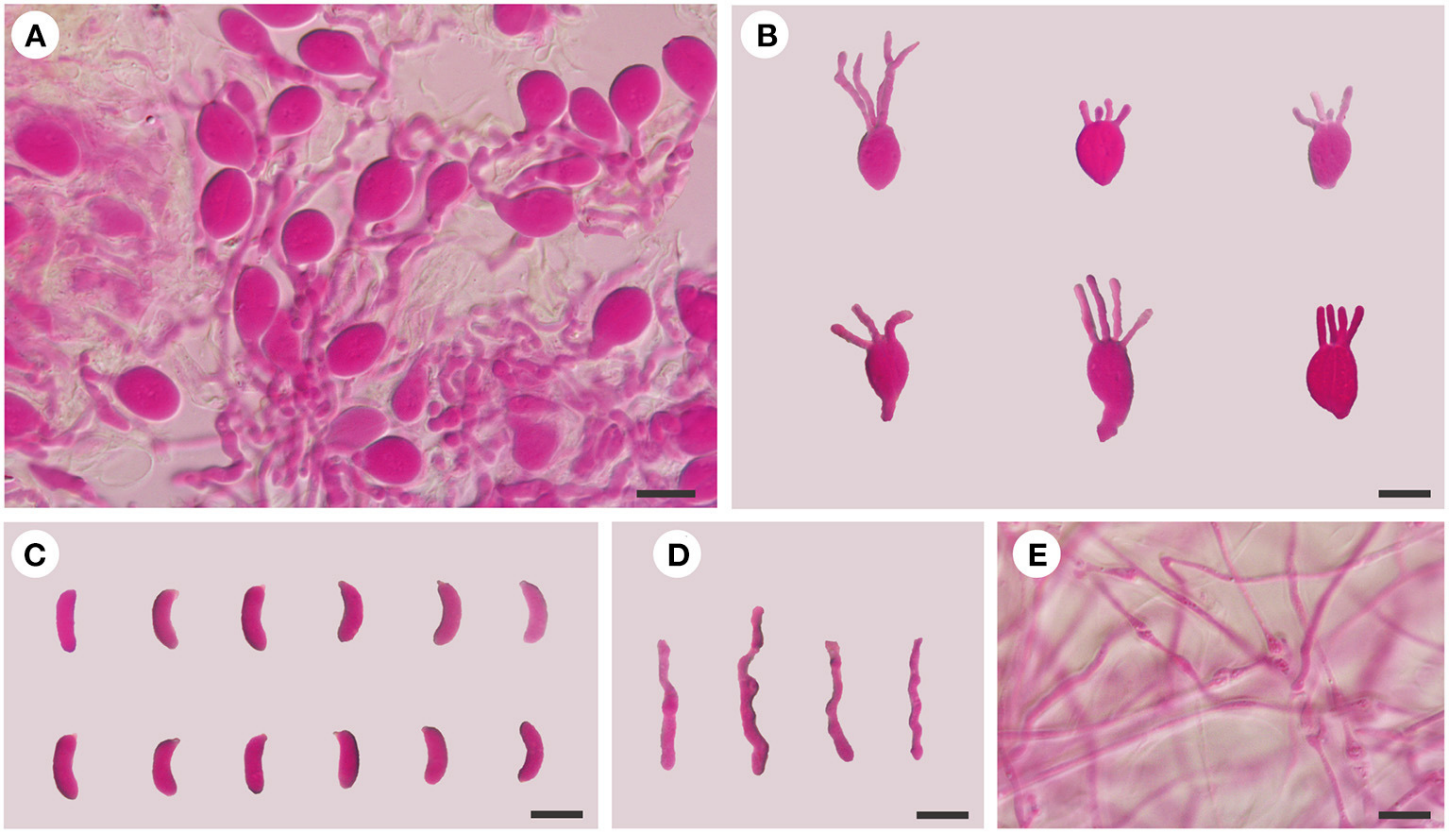
Microscopic structures of *E. saccharina* (Dai 21720). **(A)** A section of hymenium; **(B)** basidia; **(C)** basidiospores; **(D)** hyphidia; **(E)** hyphae. Scale bars: 10 μm.

*Basidiomata:* When fresh, the basidiomata are gelatinous, fawn to orange-brown, suborbicular to cerebriform, sessile, usually remaining separate, occasionally coalescing; are up to 10 cm wide and 1.5 cm thick; have free margins; have a hymenial surface that is clearly ridged, with sparse papillae, becoming vinaceous brown when dry; and are absent of mineral inclusions.

*Internal features:* Hyphal structure is monomitic; hyphae are clamped (clamps are usually open), usually branched, hyaline, thin-walled, 0.5–2.5 μm in diameter, and embedded in a gelatinous matrix. Basidia are longitudinally cruciate septate, 4-celled, subglobose to ovoid, and thin-walled, measuring 13–15.5 × 8.5–11.8 μm. Hyphidia are simple, thin-walled, and hyaline. Basidiospores are narrowly allantoid, slightly to distinctly curved, hyaline, thin-walled, smooth, usually without oil drop, neither amyloid, dextrinoid, nor cyanophilous, measuring (9.8–)10–14.2(−14.5) × (3–)3.2–4.5 μm, L = 11.71 μm, W = 3.82 μm, and Q = 2.98–3.12 (*n* = 60/2).

*Specimens examined*: CHINA. Hebei Province, Weichang County, Saihanba National Forest Park, on fallen trunk of *Larix*, 27.VIII.2020, Dai 21719 (BJFC 035621), and Dai 21720 (BJFC 035622); Xinjiang Autonomous Region, Burjin County, Karnas Nature Reserve, on fallen trunk of *Larix*, 11.IX.2015, Dai 15890 (BJFC 019991); Habahe County, Baihaba River Forest Park, on the fallen trunk of *Larix*, 10.IX.2015, Dai 15848 (BJFC 019949).

**Figure 4:**
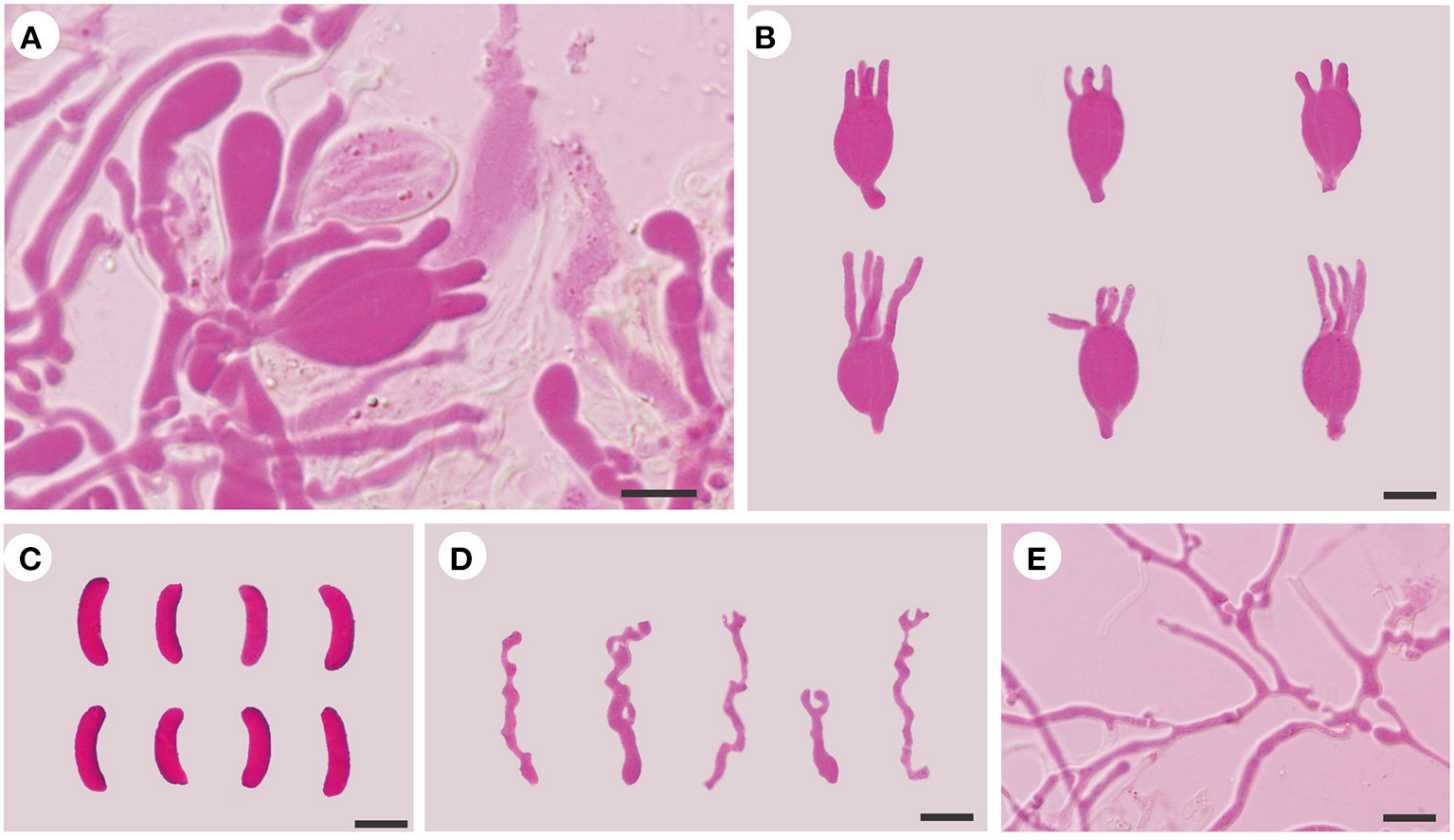
Microscopic structures of *E. subsaccharina* (Dai 22187 and LY BR 337, holotype). **(A)** A section of hymenium; **(B)** basidia; **(C)** basidiospores; **(D)** hyphidia; **(E)** hyphae. Scale bars: 10 μm.

***Exidia subsaccharina*** F. Wu, B. Rivoire, A. Tohtirjap, and Y.C. Dai, sp. nov. Figures 2B, 4.

*MycoBank*: MB846784.

*Holotype*: FRANCE. Chaussan, on dead tree of *Pinus sylvestris*, 10.VIII.2008, Dai 22187 and LY BR 337 (BJFC 036778).

*Etymology*: *Subsaccharina* (Latin) refers to the micromorphology being similar to that of *E. saccharina*.

*Diagnosis: E. subsaccharina* may be confused with *E. saccharina* when fresh, but *E. saccharina* differs from the species by its slightly smaller basidia (13–15.5 × 8.5–11.8 μm), usually simple hyphidia, and distinctly smaller basidiospores (10–14.2 × 3.2–4.5 μm).

*Basidiomata:* When fresh, the basidiomata are gelatinous, reddish brown to vinaceous brown, orbicular to suborbicular, sessile, usually remaining coalescing, occasionally separate; are fused together with up to 10 cm in width and 1 cm in thickness; have free margins; have a hymenial surface that is slightly ridged, with papillae, becoming fuscous when dry; and are absent of mineral inclusions.

*Internal features:* Hyphal structure is monomitic; hyphae are clamped (clamps are usually open), usually branched, hyaline, thin-walled, 0.5–3 μm in diameter, and embedded in a gelatinous matrix. Basidia are longitudinally cruciate septate, 4-celled, subglobose to ovoid, and thin-walled, measuring 13.5–19.2 × 9.2–14.2 μm. Hyphidia are usually branched, sometimes simple, thin-walled, and hyaline. Basidiospores are narrowly allantoid, slightly to distinctly curved, hyaline, thin-walled, smooth, usually without oil drop, neither amyloid, dextrinoid, nor cyanophilous, measuring (12–)12.5–17.5(−18.5) × (4–)4.2–5.5(−5.8) μm, L = 15.78 μm, W = 4.73 μm, and Q = 3.23–3.44 (*n* = 60/2).

*Additional specimen examined (paratype)*: FRANCE. Orliénas, on dead tree of *Pinus sylvestris*, 22.XII.2011, Dai 22195 and LY BR 4290 (BJFC 036786).

***Tremellochaete atlantica*** Alvarenga, in Phookamsak et al., Fungal Diversity 95: 242 (2019) Figures 2C, 5.

**Figure 5:**
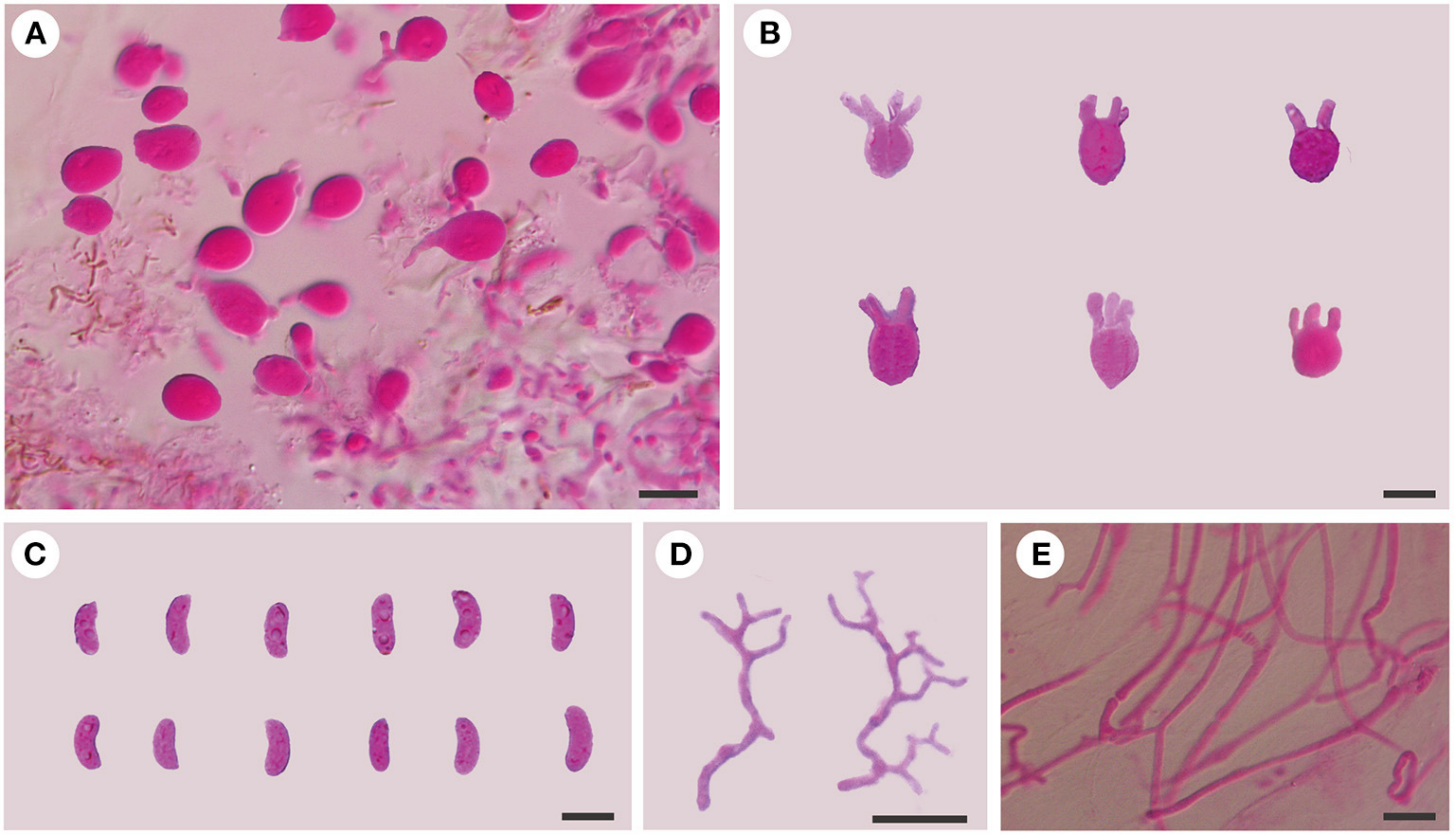
Microscopic structures of *T. atlantica* (Dai 23375). **(A)** A section of hymenium; **(B)** basidia; **(C)** basidiospores; **(D)** hyphidia; **(E)** hyphae. Scale bars: 10 μm.

*Basidiomata:* When fresh, the basidiomata are gelatinous, white to ash-gray or brownish, suborbicular to slightly cerebriform, sessile, usually remaining coalescing, occasionally separate; are fused, with up to 10 cm width and 1 cm thickness; have free margins; have occasionally ridged hymenial surface, are obviously and densely studded with irregular papillae, becoming dark gray or grayish brown when dry; and are absent of mineral inclusions.

*Internal features:* Hyphal structure is monomitic; hyphae are usually simple septate, rarely clamped, branched, hyaline, thin-walled, 0.5–2 μm in diameter, and embedded in a gelatinous matrix. Basidia are longitudinally cruciate septate, 4-celled, subglobose to globose, and thin-walled, measuring 10–12.8 × 9–10.8 μm. Hyphidia are distinctly branched, thin-walled, and hyaline. Basidiospores are allantoid, slightly to distinctly curved, hyaline, thin-walled, smooth, usually with oil drop, neither amyloid, dextrinoid, nor cyanophilous, measuring (9.8–)10–11.8(−12.8) × (3.8–)4–4.8(−5) μm, L = 10.95 μm, W = 4.24 μm, and Q = 2.58 (*n* = 30/1).

*Specimens examined*: CHINA. Fujian Province, Yongtai County, Tianmenshan National Forest Park, on fallen angiosperm branch, 5.VI.2021, Dai 22363 (BJFC 036947) and Dai 22375 (BJFC 036959); Yunnan Province, Xishuangbanna, Mengla County, Rainforest Valley Scenic Area, on fallen angiosperm branch, 3.VII.2021, Wu 539 (BJFC 036394).

**Figure 6:**
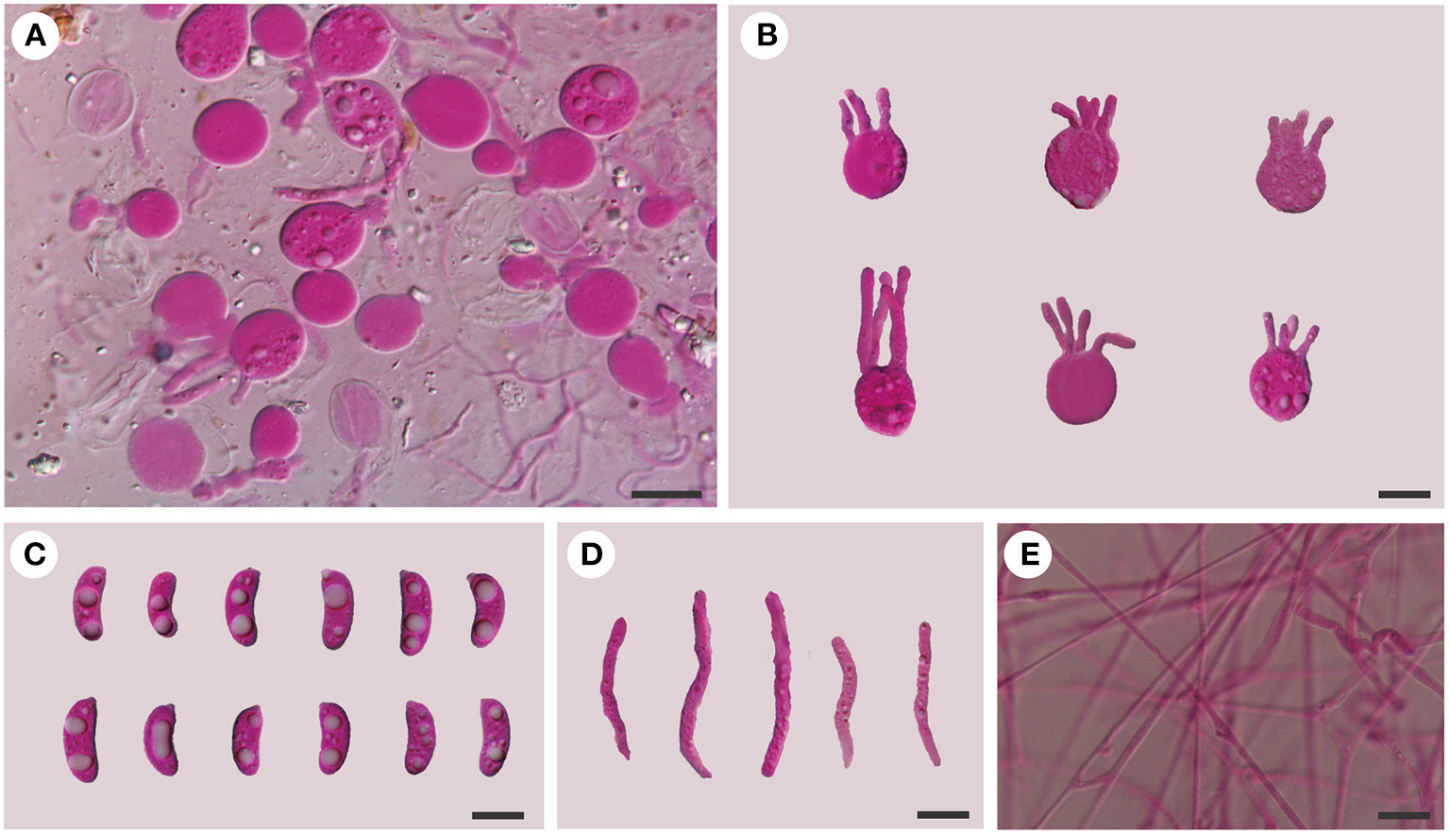
Microscopic structures of *Tremellochaete australiensis* (Dai 18664, holotype). **(A)** A section of hymenium; **(B)** basidia; **(C)** basidiospores; **(D)** hyphidia; **(E)** hyphae. Scale bars: 10 μm.

***Tremellochaete australiensis*** F. Wu, G.M. Gates, A. Tohtirjap, and Y.C. Dai, sp. nov. Figures 2D, 6.

*MycoBank*: MB846785.

*Holotype*: AUSTRALIA. Melbourne, Dandenong Ranges Botanical Garden, on the dead tree of *Rhododendron*, 12.V.2018, Dai 18664 (BJFC 027132).

*Etymology*: *Australiensis* (Latin) refers to the species being found in Australia.

*Diagnosis: Tremellochaete australiensis* is morphologically similar to *T. atlantica* and *T. japonica*, but the latter two species have shorter basidia (< 13 μm in length) and basidiospores (< 12 μm in length) and branched hyphidia.

*Basidiomata:* When fresh, the basidiomata are gelatinous, white to grayish blue, suborbicular to slightly cerebriform, sessile, usually remaining coalescing, occasionally separate; are fused together with up to 20 cm in width and 0.5–1 cm in thickness; have free margins; have a hymenial surface occasionally ridged, is clearly and densely studded with irregular papillae, becoming dark gray to black when dry; and are absent of mineral inclusions.

*Internal features:* Hyphal structure is monomitic; hyphae are clamped (clamps are usually open), usually branched, hyaline, thin-walled, 0.5–2.5 μm in diameter, and embedded in a gelatinous matrix. Basidia are longitudinally cruciate septate, 4-celled, subglobose to globose, and thin-walled, measuring 13–15.8 × 11.5–15 μm. Hyphidia are simple, cylindrical, thin-walled, and hyaline. Basidiospores are allantoid, slightly to distinctly curved, hyaline, thin-walled, smooth, usually with oil drop, neither amyloid, dextrinoid, nor cyanophilous, measuring (12.8–)13.8–16.2(−18) × (4.5–)4.8–6.5 μm, L = 14.94 μm, W = 5.59 μm, and Q = 2.6 (*n* = 30/1).

*Additional specimens examined (paratypes)*: AUSTRALIA. Tasmania, Hobart, Mt. Wellington, on rotten wood of *Olearia*, 13.V.2018, Dai 18704 (BJFC 027173) and Dai 18714 (BJFC 027183); Mount Field Forest, close to Mount National Park, on the fallen trunk of *Nothofagus*, 14.V.2018, Dai 18758 (BJFC 027226); Victoria, Yarra Ranges National Park, on rotten wood of *Eucalyptus*, 9.V.2018, Dai 18601A (BJFC 027070).

## Discussion

*Exidia sensu lato* is a genus of wood-inhabiting fungi that grows on dead branches and logs and is best known in the temperate regions of Europe, America, and Asia (Malysheva, 2012; Spirin et al., 2018; Wu et al., 2020a; Ye et al., 2020; Wang and Thorn, 2021). Although nearly eighty taxa were recorded in *Exidia sensu lato*, most species were described in the 20th century (Fries, 1822; Lowy, 1964, 1971). In recent years, four species in *Exidia* and two species in *Tremellochaete* were described based on morphology and phylogenetic analyses (Alvarenga et al., 2019; Wu et al., 2020a; Ye et al., 2020; Wang and Thorn, 2021), which improved knowledge of *Exidia sensu lato* across the world. However, since the demarcation of *Exidia* and *Tremellochaete* is still ambiguous, multilocus analyses based on taxonomically and geographically broad sampling are needed.

*Tremellochaete* was accepted by most researchers (Malysheva and Spirin, 2017; Malysheva et al., 2018; Yuan et al., 2018; Alvarenga et al., 2019). However, Wang and Thorn (2021) rejected *Tremellochaete* because its type species *T. japonica* was closely related to *E. candida* Lloyd in their phylogeny. In our phylogeny (Figure 1), three *Tremellochaete* species, *T. atlantica, T. australiensis*, and *T. japonica*, are also closely related to *E. candida*, but they formed a separate clade with robust support; two other species placed in *Tremellochaete, T. cerradensis* and *T. ciliata*, are distantly related (Figure 1). *Tremellochaete* may be a polyphyletic genus like other genera, e.g., *Exidia* and *Exidiopsis* (Yuan et al., 2018; Alvarenga et al., 2019; Spirin et al., 2019b). In addition, *Tremellochaete* can be distinguished from *Exidia* by its clear and dense papillae on the hymenial surface (Malysheva and Spirin, 2017; Alvarenga et al., 2019; Figure 2).

*Exidia subsaccharina* is morphologically similar to *E. saccharina* by sharing gelatinous and brownish basidiomata, a slightly papillate hymenial surface, and narrowly allantoid basidiospores usually without oil drop and grows on rotten conifer wood (Spirin et al., 2018), and both species are closely related in the phylogeny (Figure 1). However, *E. subsaccharina* can be distinguished from *E. saccharina* by its slightly larger basidia (13.5–19.2 × 9.2–14.2 vs. 13–15.5 × 8.5–11.8 μm), usually branched hyphidia (usually simple in *E. saccharina*), and distinctly bigger basidiospores (12.5–17.5 × 4.2–5.5 vs. 10–14.2 × 3.2–4.5 μm), and they form two distinct lineages with robust support (Figure 1). *Exidia pithya* (Alb. and Schwein.) Fr. usually grows on conifer wood too, but it differs from *E. subsaccharina* by its resupinate and black basidiomata and distinctly smaller basidiospores (10–13 × 3–5 μm; Malysheva, 2012), and it is distantly related to *E. subsaccharina* in the phylogeny (Figure 1).

*Tremellochaete australiensis* may be confused with *T. atlantica* and *T. japonica* due to their gelatinous and white to gray basidiomata, densely papillated hymenial surface, and allantoid basidiospores usually with oil drop (Phookamsak et al., 2019; Ye et al., 2020), but it has longer basidia (13–15.8 × 11.5–15 μm in *T. australiensis*; 10–12.8 × 9–10.8 μm in *T. atlantica*; 9.4–12.4 × 9.1–14.2 μm in *T. japonica*) and basidiospores (13.8–16.2 × 4.8–6.5 μm in *T. australiensis*; 10–11.8 × 4–4.8 μm in *T. atlantica*; 9.4–11.8 × 3.5–4.2 μm in *T. japonica*). Furthermore, *T. australiensis* usually has cylindrical hyphidia, but they are distinctly branched in *T. atlantica* and *T. japonica*.

*Tremellochaete atlantica* was originally described from Brazil by Phookamsak et al. (2019), and our Chinese samples and the type of *T. atlantica* share almost the same ITS sequences, with < 2 base pair differences in the ITS region between the Chinese samples and the type of *T. atlantica*. The morphology of the Chinese samples fits the descriptions of *T. atlantica* except for slightly longer basidiospores (10–11.8 × 4–4.8 vs. 7.75–10 × 2–5 μm) and usually simple septate hyphae, with Brazillian specimens usually having clamped hyphae) (Phookamsak et al., 2019). These minor differences are considered intraspecific, so we consider this the first report of *T. atlantica* from China.

## Data availability statement

The datasets presented in this study can be found in online repositories. The names of the repository/repositories and accession number(s) can be found in the article/supplementary material.

## Author contributions

FW and Y-CD coordinated the project, designed the experimental plan, and acquired funding. AT and FW analyzed the data and prepared the original draft. BR and Y-CD collected the samples from the field. S-XH, GG, BR, and Y-CD reviewed and edited the manuscript. All authors contributed to the study and approved the submitted version.

## References

[B1] Alvarenga R. L. M. Spirin V. Malysheva V. Gibertoni T. B. Larsson K. H. (2019). Two new genera and six other novelties in *Heterochaete* sensu lato (Auriculariales, Basidiomycota). Botany 97, 439–451. 10.1139/cjb-2019-0046

[B2] Chen H. Y. Bao D. P. Yang R. H. Wang Y. Gao Y. Y. Li Y.. (2019). Amino acid profile and protein quality of *Exidia* sp. Acta Agric.Nucl. Sin. 33, 81–87.

[B3] Fries E. M. (1822). Systema. Mycologicum. 2, 1–274.

[B4] Hall T. A. (1999). Bioedit: a user-friendly biological sequence alignment editor and analysis program for Windows 95/98/NT. Nucleic Acids Symp. Ser. 41, 95–98.

[B5] Katoh K. Standley D. M. (2013). MAFFT multiple sequence alignment software version 7: improvements in performance and usability. Mol. Biol. Evol. 30, 772–780. 10.1093/molbev/mst010 23329690 PMC3603318

[B6] Liu B. (1992). Flora of Fungorum Sinicorum, vol. 2 (Tremellales and Dacrymycetales). Beijing: Science Press. p. 93.

[B7] Łopusiewicz Ł. (2018). Isolation, characterisation and biological activity of melanin from *Exidia nigricans*. World Sci. News 91, 111–129.

[B8] Lowy B. (1964). New species of Tremellales from Guatemala. J. Elisha Mitchell sci. Soc. 80, 65–70.

[B9] Lowy B. (1971). Flora Neotropica Monograph 6 (Tremellales). New York: Hafner Publishing Co., Inc. 153.

[B10] Maddison W. P. Maddison D. R. (2017). Mesquite: a Modular System for Evolutionary Analysis, version 3.2. Available online at: http://mesquiteproject.org

[B11] Malysheva V. (2012). A revision of the genus *Exidia* (Auriculariales, Basidiomycota) in Russia. Mikol. Fitopat. 46, 365–376.

[B12] Malysheva V. Spirin V. (2017). Taxonomy and phylogeny of the Auriculariales (Agaricomycetes, Basidiomycota) with stereoid basidiocarps. Fungal Biol. 121, 689–715. 10.1016/j.funbio.2017.05.001 28705397

[B13] Malysheva V. Spirin V. Miettinen O. Motato-Vásquez V. Hernawati Seelan, J. S. S.. (2018). Revision of *Protohydnum* (Auriculariales, Basidiomycota). Mycol. Prog. 17, 805–814. 10.1007/s11557-018-1393-635903469

[B14] Nylander J. A. A. (2004). *MrModeltest v2*. Program Distributed by the Author. Uppsala: Uppsala University, Evolutionary Biology Centre.

[B15] Petersen J. H. (1996). The Danish Mycological Society's colour-chart. Foreningen til Svampekundskabens Fremme (Greve), 1–6.

[B16] Phookamsak R. Hyde K. D. Jeewon R. Bhat J. Jones E. B. Maharachchikumbura S. S.. (2019). Fungal diversity notes 929–1035: taxonomic and phylogenetic contributions on genera and species of fungi. Fungal Divers. 95, 1–273. 10.1007/s13225-019-00421-w34899100

[B17] Posada D. Crandall K. A. (1998). Modeltest: testing the model of DNA substitution. Bioinformatics. 14, 817–818. 10.1093/bioinformatics/14.9.817 9918953

[B18] Rambaut A. (2012). Molecular Evolution, Phylogenetics and Epidemiology. FigTree ver. 1.4 Software. Available online at: http://tree.bio.ed.ac.uk/software/figtree/ (accessed 7 August, 2022).

[B19] Roberts P. (1998). A revision of the genera *Heterochaetella, Myxarium, Protodontia* and *Stypella* (Heterobasidiomycetes). Mycotaxon 69, 209–248.

[B20] Roberts P. (2001). A key to British *Exidia* species. Field Mycol. 2, 134–135. 10.1016/S1468-1641(10)60535-X

[B21] Roberts P. (2009). *Exidia nigricans*: a new and legitimate name for *Exidia plana*. Mycotaxon 109, 219–220. 10.5248/109.219

[B22] Ronquist F. Teslenko M. Van Der Mark P. Ayres D. L. Darling A. Höhna S.. (2012). MrBayes 3.2: efficient Bayesian phylogenetic inference and model choice across a large model space. Syst. Biol. 61, 539–542. 10.1093/sysbio/sys029 22357727 PMC3329765

[B23] Spirin V. Malysheva V. Larsson K. H. (2018). On some forgotten of *Exidia* and *Myxarium* (Auriculariales, Basidiomycota). *Nord. J. Bot*. 36, e01601. 10.1111/njb.01601

[B24] Spirin V. Malysheva V. Miettinen O. Alvarenga R. L. M. Gibertoni T. B. Ryvarden L.. (2019b). On *Protomerulius* and *Heterochaetella* (Auriculariales, Basidiomycota). Mycol. Prog. 18, 1079–1099. 10.1007/s11557-019-01507-023709572

[B25] Spirin V. Malysheva V. Roberts P. Trichies G. Savchenko A. Larsson K. H. (2019a). A convolute diversity of the Auriculariales (Agaricomycetes, Basidiomycota) with sphaeropedunculate basidia. Nord. J. Bot. 37, e02394. 10.1111/njb.02394

[B26] Stalpers J. Redhead S. May T. W. Rossman A. Y. Crouch J. A. Cubeta M. A.. (2021). Competing sexual-asexual generic names in Agaricomycotina (Basidiomycota) with recommendations for use. IMA Fungus 12, 1–31. 10.1186/s43008-021-00061-3 34380577 PMC8359032

[B27] Stamatakis A. (2014). RAxML version 8: a tool for phylogenetic analysis and post-analysis of large phylogenies. Bioinformatics 30, 1312–1313. 10.1093/bioinformatics/btu033 24451623 PMC3998144

[B28] Swofford D. L. (2002). PAUP^*^*: Phylogenetic Analysis Using Parsimony (*^*^ *and Other Methods); Version 4.0b10; Sinauer Associates*. Sunderland, MA, USA: Sinauer Associates.

[B29] Vilgalys R. Hester M. (1990). Rapid genetic identification and mapping of enzymatically amplified ribosomal DNA from several *Cryptococcus* species. J. Bacteriol. 172, 4238–4246. 10.1128/jb.172.8.4238-4246.1990 2376561 PMC213247

[B30] Wang S. R. Thorn R. G. (2021). *Exidia qinghaiensis*, a new species from China. Mycoscience 62, 212–216. 10.47371/mycosci.2021.03.002 PMC9157777 37091320

[B31] Weiß M. Oberwinkler F. (2001). Phylogenetic relationships in Auriculariales and related groups–hypotheses derived from nuclear ribosomal DNA sequences. Mycol. Res. 105, 103–415. 10.1017/S095375620100363X

[B32] Wells K. Bandoni R. J. Lim S. R. Berbee M. L. (2004). “Observations on some species of *Myxarium* and reconsideration of the Auriculariaceae and Hyaloriaceae (Auriculariales),” in Frontiers Basidiomycete Mycol. Eching: IHW-Verlag. eds, Agerer, R., Piepenbring, and M., Blanz, P. p. 237–248.

[B33] White T. J. Bruns T. Lee S. Taylor J. (1990). Amplification and Direct Sequencing of Fungal Ribosomal RNA Genes for Phylogenetics. PCR protocols: a guide to methods and applications. New York, NY: Academic Press. p. 315–322.

[B34] Wu F. Chen J. J. Ji X. H. Vlasák J. Dai Y. C. (2018). Phylogeny and diversity of morphologically similar polypore genera *Rigidoporus, Physisporinus, Oxyporus* and Leucophellinus. Mycologia 109, 749–765. 10.1080/00275514.2017.1405215 29336678

[B35] Wu F. Man X. W. Tohtirjap A. Dai Y. C. (2022a). A comparison of polypore funga and species composition in forest ecosystems of China, North America, and Europe. For. Ecosyst. 9, 100051. 10.1016/j.fecs.2022.100051

[B36] Wu F. Tohtirjap A. Fan L. F. Zhou L. W. Alvarenga R. L. M. Gibertoni T. B.. (2021). Global diversity and updated phylogeny of *Auricularia* (Auriculariales, Basidiomycota). *J. Fungi* 7, 933. 10.3390/jof7110933 34829220 PMC8625027

[B37] Wu F. Yuan Y. Chen J. J. Cui B. K. Zhou M. Dai Y. C. (2020b). Terrestriporiaceae fam. nov., a new family of Russulales (Basidiomycota). Mycosphere 11, 2755–2766. 10.5943/mycosphere/11/1/2133622540

[B38] Wu F. Zhao Q. Yang Z. L. Ye S. Y. Rivoire B. Dai Y. C. (2020a). *Exidia yadongensis*, a new edible species from East Asia. Mycosystema 39, 1203–1214. 10.13346/j.mycosystema.200205

[B39] Wu F. Zhou L. W. Vlasák J. Dai Y. C. (2022b). Global diversity and systematics of Hymenochaetaceae with poroid hymenophore. Fungal Diver. 113, 1–192. 10.1007/s13225-021-00496-4

[B40] Ye S. Y. Zhang Y. B. Wu F. Liu H. X. (2020). Multi-locus phylogeny reveals two new species of *Exidia* (Auriculariales, Basidiomycota) from China. Mycol. Prog. 19, 859–868. 10.1007/s11557-020-01601-8

[B41] Yuan H. S. Lu X. Decock C. (2018). Molecular and morphological evidence reveal a new genus and species in auriculariales from tropical China. Mycokeys 35, 27–39. 10.3897/mycokeys.35.25271 30622400 PMC6021479

